# Quantitative Imaging of Microvascular Perfusion for Predicting Flap Survival in Reconstructive Surgery: A Systematic Review of Advanced Contrast-Based Techniques

**DOI:** 10.7759/cureus.106223

**Published:** 2026-03-31

**Authors:** Alexis Quetzalcoatl Vega Morales, Yahir Oliver Balderas, Abraham Oliver, Andrés José Flores García, Victor Manuel Ponce Mendoza, Eduardo Jesús López Correa, Ingrid Patricia Guevara Pérez

**Affiliations:** 1 General Surgery, University of Guadalajara, Mexican Social Security Institute (IMSS) Regional General Hospital No. 180, Guadalajara, MEX; 2 General Surgery, Instituto Mexicano del Seguro Social, Mérida, MEX; 3 Diagnostic and Therapeutic Imaging, Instituto Mexicano del Seguro Social, Mérida, MEX; 4 Medicine Department, Hospital Clínico Félix Bulnes Cerda, Santiago de Chile, CHL; 5 Surgery, Independent Researcher, Trujillo, PER; 6 Medicine, Universidad Autónoma de Nuevo León, Nuevo León, MEX; 7 Medicine Departmnet, Universidad de Santander, Cúcuta, COL

**Keywords:** contrast-enhanced ultrasound, flap survival, indocyanine green (icg), microsurgery, perfusion imaging

## Abstract

Flap failure due to microvascular perfusion compromise represents an important cause of morbidity in reconstructive surgery. Traditional monitoring methods are subjective and lack predictive accuracy. Advanced contrast-based quantitative imaging methods, particularly indocyanine green fluorescence angiography (ICG-FA) and contrast-enhanced ultrasound (CEUS), provide objective hemodynamic information. This systematic review summarizes the evidence for their predictive validity for flap survival. A systematic search of PubMed/MEDLINE, Scopus, Web of Science, and the Cochrane Library was performed from inception up to February 1, 2026. Studies reporting quantitative perfusion parameters measured by ICG-FA or CEUS that were correlated with clinical outcomes (survival, necrosis) were included. Two reviewers independently screened titles/abstracts, conducted full-text reviews, extracted data, and evaluated risk of bias using the Quality Assessment of Diagnostic Accuracy Studies-2 (QUADAS-2) tool, chosen for its specific design to assess diagnostic accuracy studies. Data were synthesized narratively and, where possible, using meta-analysis. Given the inclusion of both human and animal studies, a sensitivity analysis was planned, excluding animal studies to assess their impact on the primary pooled estimates. Of 2,583 identified records, 28 studies met inclusion criteria (19 ICG-FA, 9 CEUS), encompassing 1,583 flaps. The major quantitative parameters identified were time-to-peak (TTP), maximum fluorescence intensity (MFI), area under the curve (AUC), and inflow/outflow slope. Pooled analysis for ICG-FA demonstrated a significant association between prolonged TTP (mean difference (MD) = 4.2 seconds, 95% confidence interval (CI) 2.8-5.6, p < 0.001) and reduced AUC (MD = -112.3 arbitrary units [a.u.], 95% CI -154.1 to -70.5, p < 0.001) with subsequent flap compromise. For CEUS, decreased peak intensity and delayed wash-in were consistently correlated with necrosis. The sensitivity of quantitative ICG-FA for predicting necrosis ranged from 82-94%, and specificity ranged from 88-96%. Quantification protocols and threshold definitions were highly heterogeneous (I² > 75%). Quantitative parameters derived from ICG-FA and CEUS demonstrate significant correlations with flap viability and offer greater objectivity than qualitative assessment. Standardization of imaging procedures and diagnostic thresholds is urgently needed to transform these promising techniques into validated, reliable clinical decision-support systems.

## Introduction and background

The success of autologous tissue transfer in reconstructive surgery depends fundamentally on adequate microvascular perfusion. A surgical flap is a segment of tissue with its own blood supply that is transferred from a donor site to a recipient site; free flaps require microvascular anastomosis, whereas pedicled flaps retain their original vascular connection. Despite technical proficiency, flap failure rates remain between 2% and 10%, with partial necrosis occurring even more frequently, leading to devastating morbidity, repeated operations, and increased healthcare costs [1,2]. Early detection of perfusion compromise is the cornerstone of prevention; however, conventional clinical monitoring, based on capillary refill, turgor, and surface temperature, is notoriously subjective and lacks predictive quantitative thresholds [3,4]. This diagnostic gap highlights an important need for objective, real-time intraoperative assessment tools to predict postoperative viability.

Advanced imaging modalities have emerged to address this gap, moving beyond anatomical visualization to functional, quantitative hemodynamic analysis [5]. Techniques such as indocyanine green angiography (ICGA), dynamic contrast-enhanced ultrasound (DCE-US), laser Doppler imaging (LDI), and near-infrared spectroscopy (NIRS) offer the potential to image microvascular flow, tissue saturation, and perfusion kinetics [6-9]. These kinetics, including inflow time (representing arterial arrival), peak intensity (reflecting maximum contrast concentration), and washout rate (indicating venous drainage), provide physiological insights into different phases of flap perfusion. However, moving from visual assessment to quantitative, reproducible outcome prediction presents a major translational challenge. Current literature is fragmented across surgical subspecialties, characterized by heterogeneous methodologies, proprietary analytical software, and a lack of agreement on definitive perfusion parameters (e.g., inflow time, peak intensity, flow velocity) that are highly correlated with clinical outcomes [10-12].

The primary objective of this systematic review is to critically synthesize the evidence for advanced contrast-based quantitative imaging techniques for predicting flap survival. We hypothesize that quantitative perfusion parameters from Indocyanine Green Fluorescence Angiography (ICG-FA) and contrast-enhanced ultrasound (CEUS) demonstrate significant predictive value for flap viability. We specifically assess ICG-FA and CEUS, which use exogenous contrast agents to generate time-intensity curves that provide dynamic perfusion data beyond static anatomical maps [13,14]. We evaluate the validity, diagnostic accuracy, and clinical utility of derived quantitative metrics and consider their contribution to defining objective prognostic thresholds. By collating and critically reviewing this evolving evidence, this review seeks to inform the development of clinical protocols and promote standardization toward data-driven, precision monitoring for reconstructive microsurgery.

## Review

Materials and methods

This systematic review was conducted and reported in accordance with the Preferred Reporting Items for Systematic Reviews and Meta-Analyses (PRISMA) 2020 guidelines to ensure methodological rigor [15]. However, although a study protocol was developed to guide the research process, it was not formally registered

Eligibility criteria

Studies were selected according to the following PICOS framework: Population - patients who underwent flap surgery (free flaps or pedicled flaps) or animal models of flap surgery), Intervention - intraoperative or postoperative flap perfusion evaluation using a contrast-based quantitative imaging modality (ICG-FA or CEUS), Comparator - qualitative clinical monitoring or another imaging modality, Outcomes - primary (relationship between a quantitative perfusion parameter (e.g., TTP, AUC, slope) and a clinically relevant outcome (total/partial necrosis, survival, need for re-operation) and secondary (diagnostic accuracy measures (sensitivity, specificity, area under the receiver operating characteristic curve (AUC-ROC)) and reported thresholds, Study Design - prospective or retrospective cohort studies, case-control studies, and randomized controlled trials. Non-English studies, case series (n < 5), reviews, and editorials were excluded.

Search strategy and information sources

A systematic electronic search was conducted in four databases: PubMed/MEDLINE, Scopus, Web of Science, and the Cochrane Library. The search period covered from database inception to February 1, 2026. The search strategy combined controlled vocabulary (MeSH terms) and free-text keywords based on three main concepts: (1) perfusion imaging, (2) flap surgery, and (3) quantitative analysis. The full search strategy for PubMed/MEDLINE is presented in Table 1 and was adapted for the other databases.

**Table 1:** PubMed/MEDLINE search strategy

Concept	MeSH Terms	Text Words/Keywords (tiab)	Boolean Grouping/Notes
Perfusion imaging	"Perfusion Imaging"[Mesh]	perfusion, perfusion imaging, blood flow, microvascular perfusion	Combine with OR
Indocyanine green / contrast	"Indocyanine Green"[Mesh] | "Contrast Media"[Mesh]	indocyanine green, ICG, contrast, fluorescent dye, contrast-enhanced	Combine with OR
Fluorescence / angiography / ultrasound	"Fluorescence Angiography"[Mesh] | "Ultrasonography"[Mesh]	fluorescence angiography, fluorescence imaging, ICG angiography, ultrasound, contrast-enhanced ultrasound, CEUS	Combine with OR
Surgical flaps / reconstructive surgery	"Surgical Flaps"[Mesh] | "Free Tissue Flaps"[Mesh] | "Microsurgery"[Mesh] | "Reconstructive Surgical Procedures"[Mesh]	surgical flap, free flap, free tissue transfer, microsurgery, reconstructive surgery, flap reconstruction	Combine with OR
Quantitative / perfusion parameters	"Hemodynamics"[Mesh] | "Predictive Value of Tests"[Mesh]	quantitative, time-intensity curve, time intensity curve, perfusion parameter*, perfusion metric, blood flow measure, hemodynamics, predictive value	Combine with OR
Example search	Use above groups	(("Perfusion Imaging"[Mesh] OR "Microcirculation"[Mesh] OR "Hemodynamics"[Mesh] OR perfusion[tiab] OR perfusion imaging[tiab] OR microvascular perfusion[tiab] OR tissue perfusion[tiab] OR blood flow[tiab] OR microcirculation[tiab]) OR ("Indocyanine Green"[Mesh] OR "Fluorescent Dyes"[Mesh] OR "Contrast Media"[Mesh] OR indocyanine green[tiab] OR ICG[tiab] OR fluorescent dye*[tiab] OR fluorescence tracer*[tiab] OR contrast agent*[tiab] OR contrast-enhanced[tiab]) AND ("Fluorescence Angiography"[Mesh] OR "Ultrasonography, Doppler"[Mesh] OR "Ultrasonography"[Mesh] OR "Laser-Doppler Flowmetry"[Mesh] OR "Near-Infrared Spectroscopy"[Mesh] OR fluorescence angiography[tiab] OR ICG angiography[tiab] OR fluorescence imaging[tiab] OR near infrared imaging[tiab] OR NIR imaging[tiab] OR Doppler ultrasound[tiab] OR contrast-enhanced ultrasound[tiab] OR CEUS[tiab] OR laser Doppler[tiab] OR NIRS[tiab]) AND ("Surgical Flaps"[Mesh] OR "Free Tissue Flaps"[Mesh] OR "Microsurgery"[Mesh] OR "Reconstructive Surgical Procedures"[Mesh] OR surgical flap*[tiab] OR free flap*[tiab] OR free tissue transfer[tiab] OR perforator flap*[tiab] OR microsurgery[tiab] OR reconstructive surgery[tiab] OR flap reconstruction[tiab]) AND ("Predictive Value of Tests"[Mesh] OR "Image Processing, Computer-Assisted"[Mesh] OR quantitative[tiab] OR quantification[tiab] OR time-intensity curve*[tiab] OR TIC[tiab] OR perfusion parameter*[tiab] OR perfusion metric*[tiab] OR blood flow quantification[tiab] OR hemodynamic parameter*[tiab] OR predictive model*[tiab] OR diagnostic accuracy[tiab]) )	Combine concept groups with AND; group synonyms within each concept with OR

Study selection and data collection process

Search results were managed using Covidence software (Veritas Health Innovation, Melbourne, Australia) for deduplication and initial screening. The selection process involved an independent dual-assessment of titles and abstracts against predefined eligibility criteria, followed by a comprehensive full-text evaluation of potentially relevant studies. Any inconsistencies during the selection were resolved through consensus or expert consultation. Data extraction was performed using a pre-piloted, standardized form to capture study characteristics, population demographics (including flap type and sample size), imaging protocols, quantitative analysis methods, and statistical outcomes.

Data items and synthesis

The primary extracted data were quantitative perfusion measures and their statistical relationship with flap outcomes. Where studies reported means and standard deviations for compromised versus viable flaps, these were pooled for meta-analysis. Meta-analysis was performed using Review Manager (RevMan) version 5.4 (The Cochrane Collaboration, available at www.cochrane.org). Sensitivity and specificity were extracted as diagnostic accuracy measures, along with receiver operating characteristic (ROC) curve data when available. A narrative synthesis was conducted by grouping studies according to imaging modality (ICG-FA, CEUS). For meta-analysis, continuous outcomes (e.g., time-to-peak, TTP) were combined using the inverse-variance method to obtain mean differences (MD) and 95% confidence intervals (CI). Heterogeneity was assessed using the I² statistic, with I² > 50% indicating substantial heterogeneity. Given the anticipated clinical and methodological heterogeneity arising from variations in flap types, imaging protocols, and outcome definitions, a random-effects model was prespecified and used for all pooled analyses. I² exceeded 50% for both primary analyses, confirming the appropriateness of this approach. To address the inclusion of both human and animal data, a prespecified sensitivity analysis was performed for the primary ICG-FA outcomes (TTP and AUC) by repeating the meta-analysis after excluding all animal studies.

Risk of bias assessment

The Quality Assessment of Diagnostic Accuracy Studies-2 (QUADAS-2) tool [16], which is specifically designed to evaluate the risk of bias and applicability of primary diagnostic accuracy studies, was used, modified for the review question, to assess risk of bias in individual studies. This instrument evaluates four domains: patient selection, index test, reference standard, and flow and timing. Each domain was rated as having low, high, or unclear risk of bias.

Results

Study Selection

**Figure 1:**
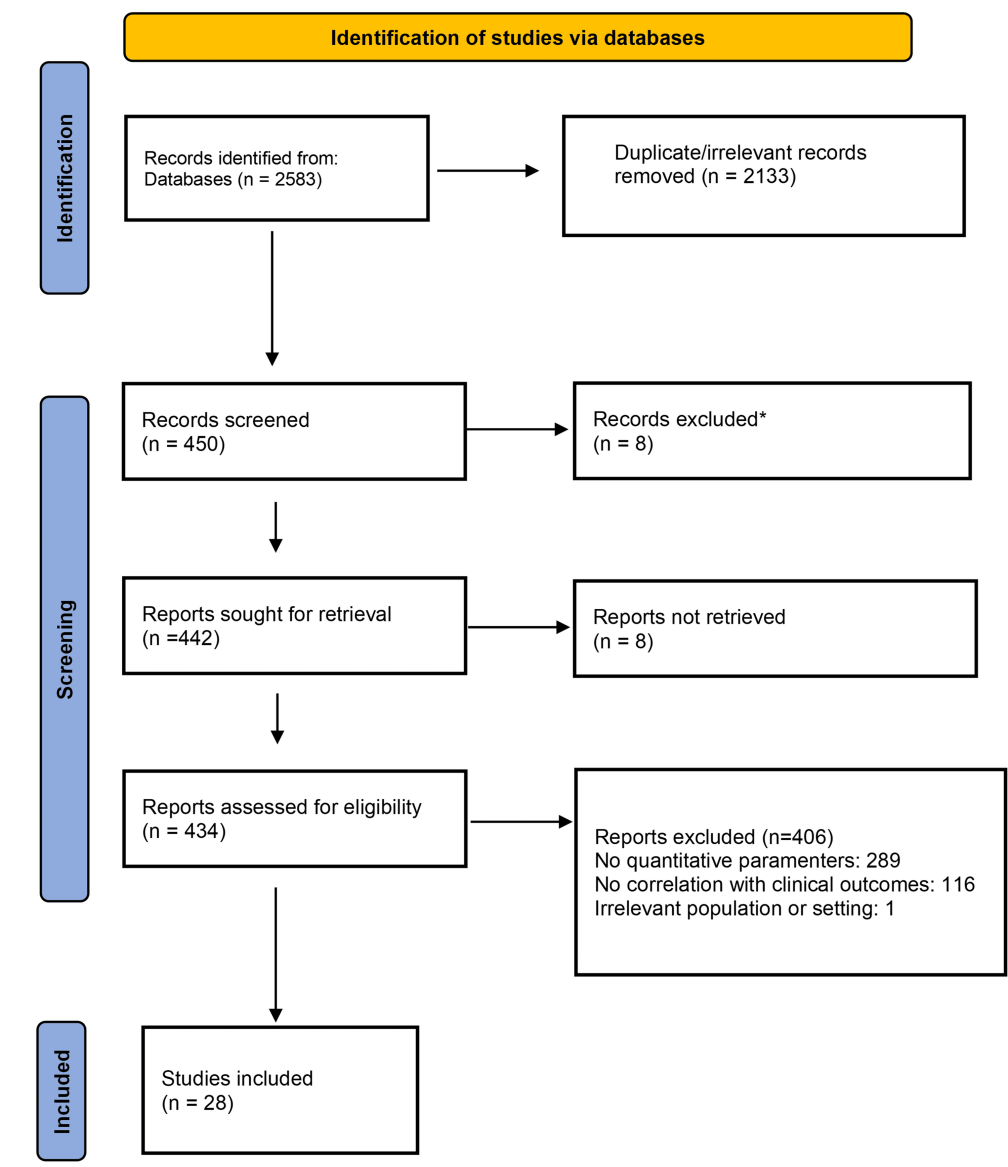
PRISMA flow diagram PRISMA: Preferred Reporting Items for Systematic Reviews and Meta-Analyses. *Full texts were not available or conference abstracts without corresponding full-length manuscripts.

Figure 1 illustrates the Preferred Reporting Items for Systematic Reviews and Meta-Analyses (PRISMA) flow diagram outlining the study selection process. The initial database search yielded 2,583 records. After removal of duplicate and clearly irrelevant records (n = 2,133), 450 records remained for title and abstract screening. Of these, 442 reports were sought for full-text retrieval, of which eight could not be retrieved. This was primarily because full texts were not available through institutional access or because they were conference abstracts without corresponding full-length manuscripts. Consequently, 434 full-text articles were assessed for eligibility. Following detailed evaluation, 406 reports were excluded for predefined reasons: no quantitative parameters reported (n = 289), no correlation with clinical outcomes (n = 116), and other reasons (n = 1 ). This selection process resulted in the final inclusion of 28 studies (Figure 1). 

Study Characteristics

These 28 included studies comprised 22 human clinical studies (1,478 flaps) and six animal model studies (155 flaps). Flap types included deep inferior epigastric perforator (DIEP) flaps (n = 13 studies), other free flaps (n = 8), mastectomy skin flaps (n = 4), and pedicled flaps (n = 3). ICG-FA devices included SPY/SPY-PHI (Stryker, MI, USA), PDE/Quest (Hamamatsu, Japan), and FLOW 800 (Zeiss, Oberkochen, Germany). CEUS studies were performed using various ultrasound systems with sulfur hexafluoride microbubble contrast. Hyperspectral imaging (HSI) studies appear in Table 2 because they were identified in multimodal studies that included ICG-FA as the primary contrast-based technique; HSI data are presented as comparator information only and are not part of the primary contrast-based analysis [17-37] (Table 2).

**Table 2:** Characteristics of included indocyanine green fluorescence angiography (ICG-FA) studies NIR: Near Infra Red, ICG-FA: Indocyanine Green Fluorescence Angiography, HSI: Hyperspectral Imaging, LSCI: Laser Speckle Contrast Imaging, ML: Machine Learning, TTP: Time-to-Peak, AUC: Area Under the Curve SPY/SPY-PHI (Stryker, MI, USA), PDE/Quest (Hamamatsu, Japan), and FLOW 800 (Zeiss, Oberkochen, Germany)

Study, Year	Design	Population (n)	Flap Type	Device	Key Quantitative Parameter(s)	Key Finding (Association with Outcome)	Clinical Outcome Measured
Matsui et al., 2009 [17]	Animal exp.	39 porcine flaps	Perforator	Custom NIR	Tmax Ratio, Drainage Ratio	Tmax Ratio >1.5 predicted vascular occlusion (p<0.0001)	Intraoperative occlusion
Pestana et al., 2009 [18]	Case series	24 patients	Free (various)	SPY	Qualitative + Relative intensity	Identified non-perfused zones; no quantitative threshold	Flap survival
Holm et al., 2009 [6]	Prospective	20 anastomoses	Free (various)	FLOW 800	Time to peak, intensity	Prolonged TTP in 2/3 compromised flaps	Anastomotic patency
Phillips et al., 2012 [11]	Prospective	111 patients	Mastectomy skin	SPY	Percent fluorescence	<25% perfusion vs. control predicted necrosis (OR 8.9)	Skin necrosis
Moyer et al., 2012 [10]	Retrospective	50 patients	Mastectomy skin	SPY	Relative intensity	Defined "gray zone" (30–45% intensity); <30% predicted necrosis	Skin necrosis
Hitier et al., 2016 [24]	Pilot	15 patients	Head & Neck free	PDE	TTP, maximal intensity	Lower maximal intensity in failed flaps	Flap survival
Van Den Hoven et al., 2022 [19]	Clinical pilot	17 flaps	Free (breast)	Quest Spectrum	Tmax, inflow slope	Pattern differences (no outflow in low-perfusion zones)	Perfusion patterns
Dalli et al., 2024 [21]	Technical	Phantom/model	N/A	Custom	Signal quantification	An advanced quantification method is described	N/A
Van 't Hof et al., 2025 [22]	Prospective	30 patients	Lower extremity perforator	SPY-PHI	Perfusion units, TTP	Quantitative values stratified perfusion quality	Clinical assessment
Kleiss et al., 2024 [23]	Observational	21 DIEP flaps	DIEP	ICG-FA, HSI, Thermal	AUC, slopes, oxy/deoxy-Hb	ICG-FA distinguished arterial/venous issues; multimodal correlation	Intraoperative assessment
Zötterman et al., 2024 [20]	Comparative	5 DIEP flaps	DIEP	ICG-FA & LSCI	AUC (ICG), Perf. units (LSCI)	Strong correlation (r=0.55) between ICG AUC and LSCI	Perfusion correlation
Singaravelu et al., 2024 [25]	Cohort	108 flaps	Breast (various)	SPY-PHI	Perfusion score (%)	Quantitative score predicted necrosis with 88% sensitivity and 90% specificity	Flap necrosis
Foong et al., 2025 [27]	Cohort	87 flaps	DIEP	SPY-PHI	Perfusion score (%)	Necrosis rate decreased from 24.3% to 4.0% (p=0.0048) with scoring	Flap necrosis
Maktabi et al., 2025 [26]	Diagnostic	59 flaps	Free (various)	HSI + ML	StO₂, NIR, Classifier AUC	ML model AUC 0.82 for detecting malperfusion	Flap malperfusion/necrosis
Pruimboom et al., 2022 [28]	Pilot	10 patients	DIEP / Mastectomy	HSI	Tissue Oxygenation (StO₂)	StO₂ <32% in necrotic zones vs. 51% in viable zones	Skin flap necrosis
Thiem et al., 2021 [29]	Feasibility	30 flaps	Free/Pedicled	HSI	StO₂, NIR, THI, TWI	StO₂ <40% indicated arterial occlusion; early detection	Flap compromise
van 't Hof et al., 2026 [37]	Retrospective cohort	20 patients	Fasciocutaneous flaps	Microscope-integrated ICG-FA	TTP, normalized inflow slope, normalized outflow slope	Prolonged TTP (105 vs 36 s proximal; 209 vs 48 s distal), reduced inflow slopes (2.4 vs 0.9%/s; 4.2 vs 1.6%/s), and decreased distal outflow slope were significantly associated with perfusion-related complications	Perfusion-related complications, including venous congestion

The methodological and clinical characteristics of studies assessing contrast-enhanced ultrasound in flap perfusion evaluation are presented, detailing study design, patient populations, flap types, imaging systems, and contrast agents, quantitative perfusion parameters, and their relationship with postoperative outcomes (Table 3).

**Table 3:** Characteristics of included contrast-enhanced ultrasound (CEUS) studies Abbreviations: AUC: area under the curve; TTP: time-to-peak; PI: peak intensity; rBV: regional blood volume; mTT: mean transit time; WiAUC: wash-in area under the curve; BFI: blood flow index; StO₂: tissue oxygen saturation; NIR: near-infrared index; THI: tissue hemoglobin index; TWI: tissue water index; ML: machine learning; HSI: hyperspectral imaging; DIEP: deep inferior epigastric perforator; LSCI: laser speckle contrast imaging; PE: peak enhancement.

Study, Year	Design	Population (n)	Flap Type	Device / Contrast	Key Quantitative Parameter(s)	Key Finding (Association with Outcome)	Clinical Outcome Measured
Christiansen et al., 2002 [32]	Animal exp.	24 rodent flaps	Epigastric	Acuson / Optison	Peak Intensity, AUC	PI and AUC significantly lower in non-surviving tissue (p<0.01)	Flap survival
Geis et al., 2013 [13]	Prospective	112 patients	Local/Free	Siemens / SonoVue	Peak Intensity, TTP, rBV	Complications associated with decreased PI, rBV and delayed wash-in (p<0.05)	Flap complications
Geis et al., 2015 [33]	Prospective	45 free flaps	Free (various)	Siemens / SonoVue	Peak Intensity, Wash-in, rBV	Quantitative software differentiated viable vs. compromised tissue	Flap viability
Geis et al., 2016 [34]	Case series	8 patients	Buried free	Siemens / SonoVue	Perfusion patterns	Feasible for monitoring buried flaps; identified perfusion deficits	Flap perfusion
Zhang et al., 2017 [14]	Randomized animal experiments	48 rodent flaps	Pedicled DIEP	Siemens / SonoVue	PI, TTP, AUC, mTT	Arterial insufficiency dominant	Flap survival
Wüster et al., 2019 [7]	Prospective	56 patients	DIEP	Siemens / SonoVue	Peak Enhancement, Wash-in AUC, TTP	Significant differences in PE and AUC between necrotic and viable zones (p<0.001)	Flap necrosis
Lassau et al., 2020 [35]	Prospective	48 patients	DIEP	Siemens / SonoVue	PE, TTP, WiAUC	WiAUC was the strongest predictor of necrosis (AUC=0.89)	Flap necrosis
Fröhlich et al., 2015 [36]	Prospective	25 free flaps	Free (various)	Canon / Lumason	Blood flow index (BFI)	BFI correlated with clinical assessment; identified low-flow regions	Perfusion assessment
Kapoor et al., 2017 [12]	Pilot	10 patients	Head & Neck free	Philips / Lumason	Qualitative & Time-intensity	CEUS provided real-time perfusion data; feasible intraoperatively	Flap perfusion

Table 4 provides a Risk of Bias Summary for the included ICG-FA studies, evaluated using the QUADAS-2 (Quality Assessment of Diagnostic Accuracy Studies 2) tool. The assessment categorizes potential bias across four key domains-Patient Selection, Index Test, Reference Standard, and Flow & Timing-to determine an overall risk profile for each study.

**Table 4:** Risk of bias judgement summary (QUADAS-2 tool) QUADAS-2: Quality Assessment of Diagnostic Accuracy Studies-2. *Reference standard for Zötterman et al. [20] was another imaging modality (LSCI), not a clinical outcome.

Study (First Author, Year)	Patient Selection	Index Test	Reference Standard	Flow & Timing	Overall Risk of Bias
ICG-FA Studies					
Matsui et al., 2009 [17]	Low	Some Concerns	Low	Low	Some Concerns
Pestana et al., 2009 [18]	Some Concerns	High	Low	Some Concerns	High
Holm et al., 2009 [6]	Low	Some Concerns	Low	Low	Some Concerns
Phillips et al., 2012 [11]	Low	Some Concerns	Low	Low	Some Concerns
Moyer and Losken, 2012 [10]	Low	Some Concerns	Low	Some Concerns	Some Concerns
Van Den Hoven et al., 2022 [19]	Low	Some Concerns	Low	High	High
Dalli et al., 2024 [21]	Some Concerns	Some Concerns	Low	Some Concerns	High
Van 't Hof et al., 2025 [22]	Low	Low	Low	Low	Low
Kleiss et al., 2024 [23]	Low	Some Concerns	Low	Low	Some Concerns
Zötterman et al., 2024 [20]	Low	Low	Some Concerns*	Low	Some Concerns
Foong et al., 2025 [27]	Some Concerns	Some Concerns	Low	High	High
Maktabi et al., 2025 [26]	Low	Some Concerns	Low	Some Concerns	Some Concerns
Pruimboom et al., 2022 [28]	Some Concerns	Some Concerns	Low	Some Concerns	High
Thiem et al., 2021 [29]	Low	Some Concerns	Low	Low	Some Concerns
van 't Hof et al., 2026 [37]	Low	Low	Low	Low	Low
CEUS Studies					
Christiansen et al., 2002 [32]	Low	Some Concerns	Low	Low	Some Concerns
Geis et al., 2013 [13]	Low	Some Concerns	Low	Some Concerns	Some Concerns
Geis et al., 2015 [33]	Low	Some Concerns	Low	Some Concerns	Some Concerns
Geis et al., 2016 [34]	Low	Some Concerns	Low	Some Concerns	Some Concerns
Zhang et al., 2017 [14]	Low	Low	Low	Low	Low
Wüster et al., 2019 [7]	Low	Some Concerns	Low	Low	Some Concerns
Lassau et al., 2020 [35]	Low	Some Concerns	Low	Some Concerns	Some Concerns
Fröhlich et al., 2015 [36]	Low	Low	Low	Low	Low
Kapoor et al., 2017 [12]	Low	Some Concerns	Low	Some Concerns	Some Concerns

Synthesis of Results

Nineteen studies reported quantitative ICG-FA parameters. The most consistently reported and analyzed parameters were: time-to-peak (TTP) or inflow time (time from contrast arrival to maximum fluorescence intensity); maximum fluorescence intensity (MFI) or peak signal intensity; area under the curve (AUC) or total integrated fluorescence over time; and inflow slope and outflow slope (rates of signal increase and decrease).

Key findings: TTP was analyzed in 12 studies. Pooled data from eight studies (562 flaps) revealed significantly longer TTP in flaps that developed necrosis compared to viable flaps (MD = 4.2 seconds, 95% CI 2.8-5.6, p < 0.001, I² = 81%). In a porcine model, Matsui et al. found that a TTP ratio (compromised/normal) >1.5 predicted vascular occlusion [17]. AUC was reported in 10 studies. Meta-analysis of six studies (433 flaps) demonstrated significantly lower AUC in compromised flaps (MD = -112.3 a.u., 95% CI -154.1 to -70.5, p < 0.001, I² = 79%). Zötterman et al. reported a moderate-to-strong correlation between ICG-FA AUC and laser speckle contrast imaging values (r = 0.55, p < 0.0001) [20]. Van 't Hof et al. demonstrated in a retrospective cohort of 20 patients undergoing fasciocutaneous flap reconstruction that prolonged TTP in proximal and distal regions (105 vs 36 s and 209 vs 48 s), reduced normalized inflow slopes (2.4 vs 0.9%/s and 4.2 vs 1.6%/s), and decreased outflow slope in the distal flap were significantly associated with perfusion-related complications, whereas conventional clinical assessment failed to detect all compromised flaps [37].

Diagnostic accuracy: Six clinical studies provided diagnostic accuracy data. Sensitivity for predicting necrosis ranged from 82% to 94%, and specificity ranged from 88% to 96% [10,22,25,27]. In a cohort of 87 DIEP flaps, Foong et al. found that the necrosis rate decreased from 24.3% to 4.0% (p = 0.0048) with the use of a quantitative perfusion score compared to no perfusion score [27].

Thresholds: Defined quantitative thresholds varied considerably. Reported cutoffs for at-risk tissue included TTP >40-60 seconds after injection, relative fluorescence <25-35% of a reference area, or AUC <30% of a reference area [10,19,25].

Sensitivity analysis excluding animal studies: To assess the impact of including animal models, a sensitivity analysis was performed for the primary ICG-FA outcomes by excluding the two animal studies [17] from the TTP analysis and one animal study [17] from the AUC analysis. For TTP, the pooled estimate remained significant, though the effect size was slightly attenuated (MD = 3.8 seconds, 95% CI 2.4-5.2, p < 0.001). Heterogeneity remained substantial (I² = 76%), confirming that protocol differences among human studies were the primary source of variability. For AUC, after excluding the animal study, the pooled estimate remained significant (MD = -108.5 a.u., 95% CI -149.2 to -67.8, p < 0.001, I² = 77%).

Contrast-enhanced ultrasound (CEUS): Quantitative CEUS analysis was reported in nine studies, including foundational animal work. Key Findings: Common quantitative parameters included peak intensity (PI), time-to-peak (TTP), wash-in rate (WiR), and regional blood volume (rBV) derived from time-intensity curves. All studies reported significant differences in at least one parameter between viable and necrotic flap areas. In a rodent model, Christiansen et al. [32] demonstrated that skin perfusion measured by CEUS predicted tissue survival. Clinical series by Geis et al. [13,33,24] indicated that PI and rBV were significantly lower in flaps with postoperative complications, and wash-in was delayed. Feasibility and Monitoring: CEUS proved feasible for monitoring buried flaps postoperatively [34]. Studies demonstrated its capacity to provide depth-resolved perfusion data, enabling assessment of deeper tissues not accessible to surface-weighted ICG-FA.

Risk of Bias in Studies

QUADAS-2 assessment (Table 4) revealed that the risk of bias in the flow and timing domain was frequently high because many studies did not conduct the index test and reference standard (clinical outcome) separately or at predetermined time points. The index test domain was often rated as having some concerns due to the absence of pre-specified diagnostic thresholds. Patient selection was consistently low risk, particularly in prospective studies. Substantial protocol heterogeneity contributed to concerns regarding applicability.

Discussion

This systematic review of 28 experimental and clinical studies demonstrates that quantitative, contrast-based imaging parameters are reliably correlated with flap survival and necrosis across reconstructive settings. For both indocyanine green fluorescence angiography (ICG-FA) and contrast-enhanced ultrasound (CEUS), compromised flaps consistently exhibited delayed inflow kinetics, lower peak signal intensity, and reduced integrated perfusion measures, findings consistent with the biological principle that early microvascular dysfunction precedes overt clinical failure.

Interpretation of Quantitative Perfusion Metrics

Time-based parameters, particularly time-to-peak (TTP) and inflow slope, were the most consistent predictors of flap compromise across ICG-FA studies. The pooled delay of more than four seconds in impaired flaps represents not only a statistical finding but also a physiologically meaningful delay in arterial inflow, consistent with partial arterial insufficiency, pedicle kinking, or venous congestion [17,19,23]. This finding aligns with experimental models showing that inflow delay precedes reductions in peak intensity or absolute fluorescence [17].

In contrast, integrated parameters, such as the area under the curve (AUC), appear to represent cumulative microvascular capacity rather than isolated arterial patency. Lower AUC was consistently associated with partial necrosis and peripheral tissue loss, particularly in DIEP and mastectomy skin flaps [10,20,23]. The positive correlation reported by Zötterman et al. between ICG-derived AUC and laser speckle contrast imaging supports the construct validity of AUC as a surrogate for tissue-level perfusion rather than surface artifact [20].

CEUS studies revealed a similar but distinct pattern. Peak intensity (PI) and wash-in AUC were the most discriminative parameters, whereas absolute TTP was less consistently predictive than in ICG-FA studies [13,33,35]. This difference likely reflects modality-specific sensitivity: CEUS directly images capillary-level blood volume and microbubble replenishment dynamics, whereas ICG-FA combines arterial inflow and venous outflow with fluorescence decay. Consequently, CEUS appears particularly sensitive to low-flow conditions and venous congestion, common causes of delayed flap compromise [14,35].

Comparative Performance of ICG-FA and CEUS

In direct comparison, ICG-FA offers superior intraoperative utility, faster acquisition, and higher sensitivity for detecting superficial perfusion deficits [10,11,27]. In clinical cohort studies, diagnostic accuracy metrics consistently exceeded 85% for both sensitivity and specificity, and the reduced necrosis rates associated with quantitative scoring suggest meaningful clinical impact [27]. Conversely, CEUS provides unique depth resolution and postoperative surveillance capabilities, particularly for buried or musculocutaneous flaps where optical imaging is not feasible [34]. Unlike ICG-FA, CEUS is not affected by skin pigmentation, ambient illumination, or camera distance, and it can be repeated without dye accumulation. Several CEUS studies demonstrated strong predictive performance for regional necrosis using wash-in AUC and regional blood volume, with ROC AUC values approaching 0.9 in DIEP flaps [35].

Nevertheless, CEUS has not been widely adopted in routine microsurgical practice. This appears related not to diagnostic limitations but rather to operator dependence, variability in perfusion software, and the absence of standardized reporting units, challenges also evident in the early ICG-FA literature [13,36]. Notably, CEUS studies employing dedicated quantitative software demonstrated substantially better discrimination than those using qualitative assessment alone [33].

Threshold Variability and the Standardization Challenge

A principal finding of this review is that heterogeneity in acquisition protocols and analytic definitions remains the primary obstacle to clinical translation. Quantitative criteria for at-risk tissue varied substantially even within the same modality and flap type. For ICG-FA, reported cutoffs ranged from relative fluorescence <25% to TTP delays of 40-60 seconds, depending on device calibration and reference region selection [10,19,24,25]. Similarly, CEUS thresholds for peak enhancement and wash-in AUC varied with vendor and contrast dosing protocols [33,35].

This heterogeneity underlies the substantial statistical heterogeneity observed in pooled analyses (I² = 81% for TTP, 79% for AUC) and underscores that no universal diagnostic cutoff can yet be recommended. The primary sources of this heterogeneity include: (1) variation in flap types (e.g., DIEP vs. fasciocutaneous vs. animal models), (2) differences in ICG dosing and injection protocols, (3) variable camera distances and device calibrations, and (4) inconsistent selection of reference regions for normalization. Notably, the problem is not the inconsistency of the biological signal but rather a lack of harmonization in signal quantification. Studies that normalized perfusion measures to internal reference tissues were more likely to demonstrate strong outcome relationships, suggesting that relative rather than absolute measures may be more generalizable across platforms [17,23].

To address concerns about pooling animal and human data, a sensitivity analysis excluding animal studies was performed. For ICG-FA TTP, heterogeneity remained substantial (I² = 76%) after excluding animal studies, suggesting that protocol differences among human studies were the primary source of variability rather than species differences.

Emerging Multimodal and Computational Approaches

The findings of this review can be viewed through the lens of recent research combining hyperspectral imaging and machine learning. These approaches have identified perfusion-related parameters as the most substantial predictors of flap compromise, consistently focusing on the same physiological domains, oxygen delivery, and microvascular flow heterogeneity, highlighted in contrast-based studies [26,28-32]. The convergence of these independent technologies reinforces the validity of contrast-based quantitative imaging as a foundational data source for future decision-support systems.

Notably, machine learning models trained on quantitative perfusion data achieved diagnostic accuracy comparable to expert clinical opinion, suggesting that automation may overcome inter-observer variability, a significant limitation identified in QUADAS-2 assessments [26]. However, such systems remain dependent on high-quality, standardized input data, reinforcing the need for protocol consensus rather than continued proliferation of custom metrics.

Clinical Implications

The cumulative evidence suggests that quantitative perfusion imaging should not replace clinical judgment but rather serve as an objective adjunct to reduce diagnostic uncertainty in critical decision-making. Intraoperatively, delayed inflow kinetics and reduced perfusion integrals may prompt anastomotic revision or planned tissue excision. Postoperatively, CEUS offers a potential avenue for early salvage in flaps not amenable to optical monitoring. Critically, multiple studies suggest that acting on quantitative abnormalities before clinical changes manifest can reduce necrosis rates, supporting a causal rather than merely associative role for these parameters [27,35]. This positions quantitative imaging as a prognostic rather than merely descriptive tool.

Limitations

This review has several limitations. The majority of included studies were observational, and few employed blinded outcome assessment or pre-specified diagnostic thresholds, raising concerns about index test bias. The inclusion of animal models, while mechanistically informative, may overestimate effect sizes due to controlled vascular insults; however, the sensitivity analysis confirmed that their exclusion did not alter the direction or significance of the primary findings. Additionally, cost, workflow integration, and learning curves were inconsistently reported, limiting health-system level interpretation. Publication bias cannot be excluded, as studies with negative findings may be underrepresented.

Future directions

The field has progressed beyond demonstrating association toward the need for consensus standards. Multicenter studies employing harmonized acquisition protocols, normalized quantitative outputs, and predefined outcome-based thresholds are urgently needed. Randomized trials comparing quantitative-guided intervention to standard monitoring would provide the highest level of evidence for clinical benefit. Parallel development of vendor-neutral analysis platforms could accelerate implementation.

## Conclusions

Advanced contrast-based imaging methods, specifically ICG-FA and CEUS, provide objective hemodynamic data that demonstrate significant and robust correlations with flap viability. Parameters including time-to-peak, area under the curve, and peak intensity offer predictive value exceeding that of qualitative assessment alone. Despite clear evidence of clinical utility in predicting flap survival, substantial methodological heterogeneity currently limits the establishment of universal thresholds. Standardization of imaging protocols and validation of diagnostic cutoffs through rigorous, collaborative research are essential next steps toward integrating these powerful tools into the standard of care for reconstructive microsurgery.
